# Serum concentrations of IGF-I/IGF-II as biomarkers of alcohol damage during foetal development and diagnostic markers of Foetal Alcohol Syndrome

**DOI:** 10.1038/s41598-018-38041-0

**Published:** 2019-02-07

**Authors:** Vicente Andreu-Fernández, Adriana Bastons-Compta, Elisabet Navarro-Tapia, Sebastian Sailer, Oscar Garcia-Algar

**Affiliations:** 1 Grup de Recerca Infancia i Entorn (GRIE), Institut d’investigacions Biomèdiques August Pi i Sunyer (IDIBAPS), Barcelona, Spain; 2 Red Salut Materno-Infantil y del Desarrollo (SAMID), Programa RETICS, Instituto Carlos III (ISCIIII), Madrid, Spain; 3 Fundación Sant Joan de Déu, Barcelona, Spain; 4 Department of Neonatology, Hospital Clínic-Maternitat, ICGON, IDIBAPS, BCNatal, Barcelona, Spain

## Abstract

Foetal Alcohol Syndrome (FAS) is the most deleterious health effect derived from alcohol consumption during pregnancy and is placed at the end of the Foetal Alcohol Spectrum Disorders (FASD). Few studies have proposed potential molecular biomarkers of physical and neurological damage associated with prenatal alcohol exposure. We prospectively recruited 55 children from 8 to 12 years old, with a prenatal assessment for ethanol exposure using meconium analysis of fatty acid ethyl esters (FAEE). The control group was established for FAEE < 2 nmol/g (n = 31) and a Prenatal Ethanol Exposure (PEE) group for FAEEs > 2 nmol/g (n = 33). Moreover, 98 children adopted from Eastern European Countries (EEC) were also recruited to evaluate FASD diagnosis comprising 31 cases with complete FAS, 42 with partial FAS, 6 with ARBD and 5 with ARND. Serum values of IGF-I and IGF-II for all children recruited were determined by immunoassay. Anthropometric and neurocognitive evaluation showed severe impairments in FAS children, moderate effects in PEE and no harmful effects in the control group with no prenatal exposure to alcohol. Analysis of IGF-I and IGF-II serum concentrations revealed that FASD from EEC as well as PEE children showed significantly lower concentrations of both IGF-I and IFG-II than the control group and reference values. Moreover, Spearman correlations showed a significant effect of IGF-I on anthropometric measurements in girls, whereas IGF-II affected the neuropsychological variables in both genders. These findings validate the use of growth factors IGF-I and IGF-II as surrogate biomarkers of damage induced by prenatal exposure to ethanol and could be used in the diagnosis of foetal alcohol spectrum disorders.

## Introduction

Alcohol is known to be the most common human teratogen and its consumption during pregnancy can cause severe adverse effects on the human foetus. The severity of foetal damage due to alcohol exposure depends substantially on the consumption pattern and the dose of alcohol.

Foetal Alcohol Spectrum Disorders (FASD) are a range of deleterious health consequences of alcohol consumption during pregnancy and the most severe result is Foetal Alcohol Syndrome (FAS)^1^. The clinical features of FAS can be broadly divided into: (1) morphological malformations, especially craniofacial features, (2) growth retardation, and (3) central nervous system (CNS) impairment, expressed mainly as learning disabilities and behavioural problems^2,3^. In the USA, the prevalence of FAS is 1.3 to 4.6 births per 1000^4^, while the prevalence of FASD is estimated to be as high as 9.1 per 1000^5^. However, in other countries such as South Africa, the prevalence of FAS ranges from 5.5% to 6.3%^6,7^. An increased risk of prenatal exposure to ethanol (PEE) and FASD has been reported in children adopted from Eastern European Countries (EEC)^8–11^. Landgren *et al*. reported a FASD prevalence of 52% in children adopted in Sweden from EEC, including Russia, Poland, Romania, Estonia and Latvia^8^. According to phenotypic characteristics, 58% of the children adopted from EEC and the former Soviet Union have a high/intermediate risk for PEE^10^. However, information about PEE is unavailable for many adoptees^9–11^. Maternal alcoholism has been specifically mentioned in the adoption records of 21% to 33% of adoptees from EEC^8–11^. Early diagnosis of FASD is important in order to mitigate secondary disabilities which arise later in life. FAS can be diagnosed correctly based on facial features and neurocognitive disabilities in school-age children, but for the majority of FASD cases, a precise strategy for diagnosis is still being researched. Detection of PEE has been focused on the use of questionnaires about alcohol consumption and biomarkers of exposure in biological matrices^12^. The determination of fatty acid ethyl esters (FAEEs) or ethyl-glucuronide (EtG) in meconium and/or maternal hair is the best procedure to identify exposed newborns. However, the cut off value to consider positive alcohol exposure is in the range of heavy drinking population^13,14^. In addition, research of accurate biomarkers to identify children with FASD is required, that is, biomarkers of damage as surrogate prenatal alcohol exposure biomarkers.

Few studies have proposed potential biomarkers of physical and neurological damage associated with prenatal alcohol exposure. Carter *et al*. have recently demonstrated that growth restriction could be used both as a diagnostic criterion and as an early FASD biomarker in children affected by PEE^15^. In this study, the authors observed that children heavily exposed to ethanol showed significant prenatal and postnatal growth restriction which correlated with a greater risk for cognitive deficits generated during foetal development. These results demonstrated that children’s growth pattern might predict the severity of neurocognitive damage produced by foetal alcohol exposure^15^. Moreover, recent work^16^ analysed the effect of PEE (identified by questionnaire) on insulin-like growth factors (serum concentrations of IGF-I and IGF-II) during infancy and childhood. The authors observed a significant decrease in IGF-I serum concentrations as well as an increase in IGF-II concentrations in children prenatally exposed to alcohol, suggesting that these growth factors could be identified as potential biomarkers of PEE^16^. However, although the decrease of IGF-I has been previously observed^17^, the significance of serum levels of IGF-II in FASD children is still controversial. A study in rodents demonstrated that chronic gestational exposure to alcohol alters gene expression of IGF signalling pathways, reducing mRNA levels of IGF-I and IGF-II^18,19^. Other evidence also suggested a possible effect of alcohol on the regulation of the IGF-I metabolism, triggering reduced levels of IGF-I, IGFBP3 but no alteration in IGF-II concentrations^17^.

In this context, the objective of this study was to determine whether IGF-I and IGF-II can be validated as biomarkers of damage produced by alcohol exposure during pregnancy.

## Results

### FASD prevalence

FASD diagnosis was assessed in 55 native children from a Meconium Project and 98 EEC adoptees. Meconium analysis of FAEE in Spanish children showed that 24 subjects had been prenatally exposed to ethanol (PEE, meconium FAEE > 2 nmol/g) and 31 were negative (meconium FAEE ≤ 2 nmol/g), which then acted as the control group. After performing clinical evaluations, two of the PEE children from the Meconium project were diagnosed with ARND.

Moreover, 84 of the EEC children were diagnosed with FASD while 11 were considered NO FASD and 3 children were diagnosed with other syndromes such as attention deficit hyperactivity disorder (ADHD). However, the adoption documents and judicial resolutions indicated that all NO FASD adoptees had suffered PEE. In the FASD group we found 31 cases with complete FAS, 42 with partial FAS, 6 with alcohol-related birth defects (ARBD) and 5 cases with alcohol-related neurodevelopmental disorder (ARND). There was no significant difference in the mean age of each EEC diagnostic group (Table 1) and no bias was found in gender distribution.

**Table 1:** Descriptive characteristics and anthropometric measurements of children population. *t-test* was used to compare the outcomes of the different groups: non-exposed children, PEE and FASD.

	Control (n = 31)	PEE (n = 33)	FASD			
			FASc (n = 31)	FASp (n = 42)	ARBD (n = 6)	ARND (n = 7)
**Descriptive characteristics**						
Gender (n, % of females)	18 (58)	12 (36)	18 (58)	12 (28)	2 (33)	4 (57)
Age (years)	10.2 (0.6)	10.4 (1.7)	9.6 (2.9)	9.7 (3.1)	10.8 (2.6)	11.2 (3.1)
**Anthropometric measurements**						
Height (cm)	141.3 (8.1)	142.3 (11.8)	**127**.**7 (14**.9)*	137.4 (17.9)	146.4 (20.4)	142.5 (19.3)
<10^th^ percentile (%)	3.4	3.0	61.3	4.8	0	28.6
Weight (kg)	36.6 (7.2)	37.4 (11)	**24**.**2 (7**.7)*	32.9 (12.5)	39.6 (14.7)	38.7 (14.5)
<10^th^ percentile (%)	0	5.9	90.3	11.9	0	0
Head circumference (cm)	54.4 (3.4)	53.5 (1.8)	**48**.**4 (2**.1)*	**50**.**9 (2**.7)*	53.2 (1.8)	**50**.**7 (2**.5)*
<10^th^ percentile (%)	10	5.9	100	64.3	0	57.1
BMI (kg/m^2)^	18.2 (2.6)	18.2 (2.9)	**14**.**5 (2**.4)*	**16**.**7 (2**.5)*	17.8 (1.9)	18.4 (2.4)
Severely underweight, <16 (%)	13.3	23.5	90	47.6	16.7	14.3

Data for age, height, weight, head circumference and BMI are represented by mean and standard deviation (SD). *p-value < 0.05 compared to control group.

Alcohol-Related Birth Defects (ARBD); Alcohol-Related Neurodevelopmental Disorder (ARND); Foetal Alcohol Spectrum Disorder (FASD); Foetal Alcohol Syndrome (FASc); partial Foetal Alcohol Syndrome (FASp); Prenatal Ethanol Exposure (PEE).

### Effect of prenatal ethanol exposure on anthropometric variables

The effects of gestational ethanol consumption on anthropometric measurements were evaluated (Table 1). Children diagnosed with complete FAS had a significant reduction in the mean of height, weight, head circumference percentile (HCP) and Body Mass Index (BMI) in comparison to the control group, which correlated with an increase in the percentage of children below the 10^th^ percentile for these parameters (61% vs 3%, 90% vs 0%, 100% vs 10%, 90% vs 10%, respectively). Partial FAS also showed a significant reduction of head circumference and BMI parameters in comparison to the control group. Moreover, children diagnosed with ARBD and ARND did not present significant rates with anthropometric variables <10^th^ percentile. Interestingly, microcephaly was significantly present in all groups of FASD except in the group of ARBD (Table 1).

### Neurocognitive evaluation for FASD diagnosis

Neurocognitive assessment revealed that a significant proportion of children with complete and partial FAS had lower scores on the four domains which composite the Full-Scale IQ, and also on the IQ compared to the control group. The most affected domains were the Working Memory Index and the full-scale IQ (FSIQ, 66% in complete FAS and 42% in partial FAS *versus* 3% in the control group).

33% of the children with complete FAS and 26% of partial FAS showed a moderate mental retardation (Table 2).

**Table 2:** Neurocognitive assessment. *t-test* analyses were performed to compare the results of FASD, PEE and non-exposed children.

	Control	PEE	FASD			
			FASc	FASp	ARBD	ARND
**Neurocognitive assessment (%)**						
Verbal comprehension Index	0	4.54	**55**.6*	**26**.3*	0	0
Perceptual Reasoning Index	0	4.54	**33**.3*	**26**.3*	0	0
Working Memory Index	3.3	18.18	**66**.7*	**42**.1*	0	0
Processing Speed Index	6.7	4.54	**33**.3*	**47**.4*	0	0
Full-scale IQ	3.3	4.54	**66**.7*	**36**.8*	0	0
Moderate mental retardation	0	4.54	**33**.3*	**26**.3*	0	0

The values of each neurocognitive Index analysed show the percentage of children with < 70 score, <2 SD (%) for each population group evaluated. *p-value < 0.05 compared to control group.

Alcohol-Related Birth Defects (ARBD); Alcohol-Related Neurodevelopmental Disorder (ARND); Foetal Alcohol Spectrum Disorder (FASD); Foetal Alcohol Syndrome (FASc); partial Foetal Alcohol Syndrome (FASp); Prenatal Ethanol Exposure (PEE).

### Effect of prenatal ethanol exposure on IGF axis

Three groups of children were analysed: FASD (considering complete FAS, partial FAS, ARND and ARBD), PEE (without a diagnosis of FASD but prenatal exposure to alcohol confirmed by FAEE presence in meconium) and controls (meconium FAEE < 2 nmol/g).

IGF-I and IGF-II serum concentrations were evaluated by commercial immunoassays. The IGF-I immunoassay (REF: DG100; R&D Systems) has a sensitivity of 0.026 ng/ml, (intra-assay CV is 4.03%, and the inter-assay CV is 7.97%). The IGF-I reference values for age and gender distribution of the children included in this study were obtained from the Nichols Institute Diagnostics^20^. The IGF-II immunoassay (REF: RMEE30; Biovendor) has a sensitivity of 0.02 ng/ml (intra-assay CV is 4.84% and the inter-assay CV is 7.11%) and it is validated for *in vitro* diagnostic use in the European Union. The IGF-II reference values were supplied by the immunoassay provider. All procedures were performed following the guidelines from Good Laboratory Practices (GLP).

Firstly, analysis of IGF-I and IGF-II values using a non-parametric analysis (Dunn’s multiple comparison test) showed significant differences between controls and PEE and also between controls and FASD (or FASc and FASp separately) for IGF-II (Table 3). For IGF-I comparisons, only females diagnosed with FASD (or FASc) demonstrated significant differences when compared to the control group. As previously reported, IGF-I shows a clear gender and age dependency^20,21^. Linear regression analysis (Table 4) corroborated the age and gender dependency for IGF-I (*p*-value < 0.001) in contrast to IGF-II. This independence of IGF-II values in relation to age and gender confirmed the results obtained in Table 3. Accordingly, age and gender dependence for IGF-I was taken into account for all analyses performed in this study.

**Table 3:** IGF-I and IGF-II serum concentrations of control children (FAEE < 2 nmol/g) *versus* exposed (PEE, meconium FAEE > 2 nmol/g) or FASD children (FASc, FASp, ARBD, ARND) using non-parametric Dunn’s multiple comparisons *test*.

	Dunn’s multiple comparisons	Gender	IGF-I *p*-value	IGF-II *p*-value
Control vs	PEE	♂	>0.99	0.030
		♀	>0.99	
	FASD	♂	0.73	<0.0001
		♀	**0**.**002**	
	FASc	♂	>0.99	0.001
		♀	**0**.**004**	
	FASp	♂	0.06	<0.0001
		♀	>0.99	
	ARBD	♂	>0.99	0.192
		♀	>0.99	
	ARND	♂	>0.99	0.301
		♀	>0.99	

Serum levels of IGF-I observed in controls were compared to the levels obtained for PEE, FASD (including FASc, FASp, ARBD and ARND), FASc, FASp, ARBD and ARND children, distributing these analyses by gender. For IGF-II, serum levels in controls were compared to the same groups previously mentioned with no differentiation by sex. Significant differences were considered when *p*-value < 0.05. Male: ♂; Female: ♀.

Alcohol-Related Birth Defects (ARBD); Alcohol-Related Neurodevelopmental Disorder (ARND); Foetal Alcohol Spectrum Disorders (FASD); Foetal Alcohol Syndrome (FASc); partial Foetal Alcohol Syndrome (FASp); Prenatal Ethanol Exposure (PEE); ns: statistically non-significant.

**Table 4:** Gender and age dependency of IGF-I and IGF-II serum concentrations evaluated by simple linear regression.

Variable	IGF-I					IGF-II				
	B	Std. Error	Beta	t	*p*-value	B	Std. Error	Beta	t	*p*-value
Age (years)	9,34	2.49	0,37	3.76	0.001	7,11	7.43	0,09	0.96	0.341
Gender	−41,90	11.88	−0,35	−3.53	0.001	7,66	38.67	0,02	0.20	0.843

Significant differences were considered when *p*-value < 0.05. Unstandardized Coefficients: B with Standard error (Std.Error); standardized coefficient: Beta, t value.

Moreover, multiple comparison analyses were performed to validate IGF-I concentrations in controls and PEE recruited children (from 8 to 12 years old) with the reference values used in the literature^20^. As can be observed in Table 5 (left side), the control group did not show significant differences when compared to reference values in the age range evaluated. In contrast, IGF-I values of PEE children showed differences compared to reference values (Table 5, right side) for both gender and age distribution.

**Table 5:** Comparison among IGF-I serum concentrations of reference values, controls (with negative exposition) and exposed children to ethanol (PEE, meconium FAEE > 2 nmol/g), using *t test* analysis.

IGF-I		Control group			PEE		
Age (years)	Gender	Mean	SD	*p*-value	Mean	SD	*p*-value
8–10	♂	98.9	5.7	0.162	85.3	11.7	0.037
	♀	170.4	86.3	0.922	114.7	19.4	0.039
10–12	♂	170.1	34.5	0.091	116.1	16.0	0.003
	♀	221.6	32.7	0.062	153.7	28.1	0.011

Significant differences were considered when *p*-value < 0.05. Male: ♂; Female: ♀.

Prenatal Ethanol Exposure (PEE); SD: Standard deviation.

**Figure 1:**
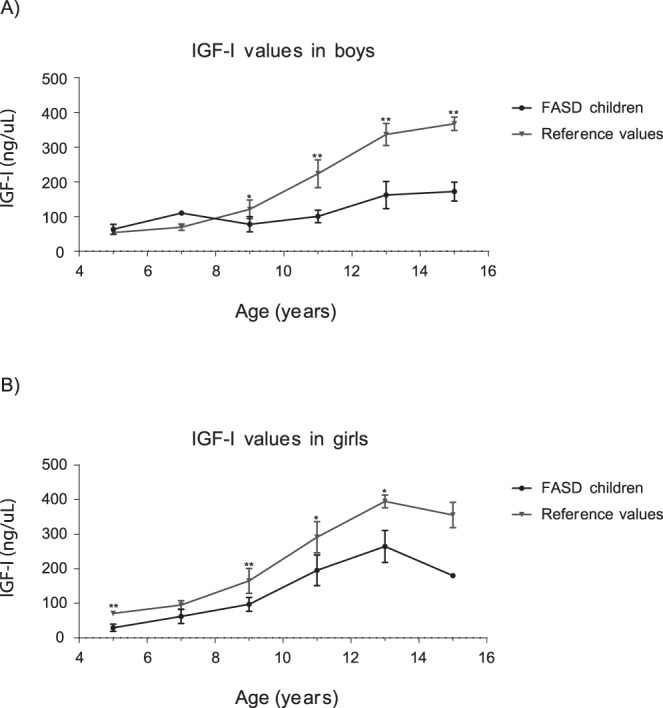
IGF-I serum concentration trends in childhood and adolescence. IGF-I averages of FASD children were compared with reference values using *t test* analyses. Each value was obtained by IGF-I immunoassay, taking into account a specific distribution for age-range (from four to sixteen) and gender. *p-value < 0.05. **p < 0.01. (**A**) IGF-I means for boys. (**B**) IGF-I means for girls.

Subsequently, IGF-I concentrations of FASD children were evaluated. Both boys and girls showed significantly lower values of IGF-I compared to reference values (Table 6). For boys (Fig. 1A), marked differences in IGF-I concentrations were observed from 8 to 16 years old. For girls, the values were significantly lower than reference values from 6 to 16 years old (Fig. 1B).

**Table 6:** Comparison between the IGF-I serum concentrations of reference values and FASD children, using *t test* analysis.

IGF-I				
Age (years)	Gender	Mean	SD	*p*-value
4–6	♂	63.4	14.3	0.3189
	♀	29.5	10.5	**0.0057**
6–8	♂	110.1	1.1	**0.0097**
	♀	62.4	20.6	0.0622
8–10	♂	77.8	21.8	**0.0273**
	♀	97.1	20.9	**0.0075**
10–12	♂	100.5	18.2	**0.0001**
	♀	195.8	44.4	**0.0113**
12–14	♂	162.2	38.9	**0.0006**
	♀	264.7	46.2	**0.0188**
>14	♂	172.2	27.3	**0.0005**

Significant differences were considered when *p*-value < 0.05. Male: ♂; Female: ♀.

SD: Standard deviation.

**Figure 2:**
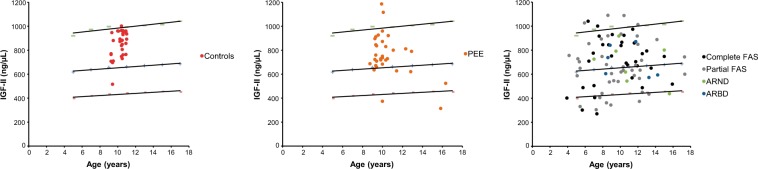
Scatter plots for IGF-II serum values in population analysed. Lines indicate thresholds of < 5^th^ (bottom) < 50^th^ (middle) and < 95^th^ (top) percentiles. Following colour spots represented the diagnosis of all children recruited in this study: Controls (meconium FAEE < 2 nmol/g, red spot), PEE (meconium FAEE > 2 nmol/g, orange spot). Complete FAS (black spots), partial FAS (grey spot), ARND (green spot) and ARBD (blue spot).

For IGF-II, the percentile < 5^th^ and < 50^th^ were calculated for control, PEE, and each category of FASD, taking into account reference values supplied by the immunoassay provider. Results for IGF-II values are showed in Fig. 2 and Table 7. Children of the control group were not represented in the 5^th^ percentile, and merely one child was included in the 50^th^ percentile. Conversely, the PEE group exhibited a proportion of 6.5% in the 5^th^ percentile and 19.4% in the 50^th^ percentile, demonstrating moderate damage resulting from ethanol exposure in these children. Then, the relationship between IGF axis and FASD diagnosis was explored, showing significant differences compared to the control and PEE groups (Table 7, bottom). Additionally, 14% of FASD children were present in the 5^th^ percentile, increasing to 43% in the 50^th^ percentile. When different categories of FASD were studied, complete and partial FAS showed a substantial presence in the 5^th^ and the 50^th^ percentile, confirming the results obtained in Table 3. For children diagnosed with ARBD or ARND, no relevant differences were observed to the control group in the 5^th^ percentile. However, these categories exhibited high presence in the 50^th^ percentile.

**Table 7:** IGF-II serum concentrations <5^th^ and <50^th^ percentiles across the different study populations. Reference values were used to categorize the <5^th^ and <50^th^ percentiles among non-exposed, PEE and FASD groups.

Differences on IGF axis based on FASD diagnosis	IGF-II < 5^th^ percentile n (%)	IGF-II < 50^th^ percentile n (%)
Control group (n = 31)	0 (0%)	1 (3%)
PEE children (n = 33)	2 (6.5%)	6 (19.4%)
FASD children (n = 86)	12 (14%)	37 (43%)
Complete FAS (n = 31)	4 (13%)	10 (32.3%)
Partial FAS (n = 42)	7 (16.7%)	21 (50%)
ARBD (n = 6)	0 (0%)	3 (50%)
ARND (n = 7)	1(14.3%)	3 (42.9%)

Alcohol-Related Birth Defects (ARBD); Alcohol-Related Neurodevelopmental Disorder (ARND); Foetal Alcohol Syndrome (FAS); Foetal Alcohol Spectrum Disorders (FASD); Prenatal Ethanol Exposure (PEE).

These results show significantly low values of IGF-I and IGF-II produced by severe damage after alcohol exposure in FASD children. These low serum concentrations, promoted by alterations in IGF metabolism, could affect different morphological and neuropsychological variables in FASD children. For that, Spearman correlations were performed as indicated in Table 8. Age dependency for IGF-I was normalized by obtaining the percentile according to age for each of the anthropometric parameters evaluated. In the case of the neuropsychological evaluation, the Wechsler intelligence scale for children (WISC-IV) was adjusted according to the age of the subjects analysed. It should be noted that ARND and ARBD were not individually analysed due to the low sample size.

**Table 8:** *Spearman correlations (r)* among IGF-I/IGF-II values and anthropometric or neurocognitive scores in the children population analysed.

Groups			Anthropometric scores				Neurocognitive scores				
			HP	WP	HCP	BMI	FSIQ	VCI	PRI	WMI	PSI
Control	IGF-I	♂	0.45	−0.31	0.57*	−0.22	0.07	0.16	0.32	−0.13	−0.12
		♀	0.33	0.43	0.12	0.20	0.60*	0.48	0.45	0.44	0.38
	IGF-II		0.25	0.34	0.14	0.26	−0.38	−0.19	−0.40	−0.31	−0.19
PEE	IGF-I	♂	0.06	0.11	0.29	0.20	0.03	0.57*	0.10	−0.15	−0.67**
		♀	0.00	0.14	0.40	0.21	−0.31	0.12	0.09	−0.20	−0.14
	IGF-II		0.08	−0.12	0.09	−0.19	0.46*	0.20	0.50**	0.42*	0.58**
FASD	IGF-I	♂	−0.12	−0.10	−0.07	0.09	−0.40	−0.47	−0.20	−0.29	−0.10
		♀	0.58**	0.45*	0.47*	0.33	0.40	−0.20	0.39	−0.20	0.40
	IGF-II		−0.15	0.04	−0.12	0.06	0.26	0.12	0.23	0.08	0.26
FASc	IGF-I	♂	−0.30	−0.40	−0.18	−0.41	—	—	—	—	—
		♀	−0.14	0.58*	0.45****	0.33	—	—	—	—	—
	IGF-II		−0.28	0.22	−0.35	0.027	−0.89*	−0.93**	−0.39	−0.41	−0.89*
FASp	IGF-I	♂	0.35	0.58*	−0.08	0.48*	0.20	−0.40	0.20	0.00	0.40
		♀	0.72**	0.15	0.41	−0.04	—	—	—	—	—
	IGF-II		−0.03	0.20	−0.34	0.19	0.79**	0.75*	0.73*	0.56	0.84**

The serum concentrations of IGF-I (or IGF-II) were compared to the values of each anthropometric and neurocognitive score for each population group evaluated (Controls, PEE, FASD, FASc, FASp). Correlations between IGF-I serum values and each of anthropometric scores or between IGF-I serum values and each of neurocognitive scores were performed distributed by sex for each population group analysed. Similar correlations were performed for IGF-II serum values with no distribution by sex. Significant differences were considered when *p*-value < 0.05 (two-tailed). *p < 0.05; **p < 0.01; ***p < 0.001; ****p < 0.0001. Male: ♂; Female: ♀.

Foetal Alcohol Spectrum Disorders (FASD); Foetal Alcohol Syndrome (FASc); partial Foetal Alcohol Syndrome (FASp); Prenatal Ethanol Exposure (PEE); ns: statistically non-significant. Anthropometric scores: Height Percentile (HP), Weight percentile (WP), Head Circumference Percentile (HCP), Body Mass Index (BMI). WISC-IV neurocognitive scores: Full Scale IQ (FSIQ), Verbal Comprehension Index (VCI), Perceptual Reasoning Index (PRI), Working Memory Index (WMI) and Processing Speed (PSI).

In contrast to the control group (with no significant correlations), children prenatally exposed to alcohol (PEE) showed a clear positive correlation among IGF-II values and the neuropsychological scores for full-scale IQ (FSIQ) (0.57**), Perceptual Reasoning Index (PRI) (0.50**), Working Memory Index (WMI) (0.42*) and Processing Speed Index (PSI) (0.58**). Similar results were obtained for FASp children, with a positive correlation for FSIQ (0.79**), Verbal Comprehension Index (VCI) (0.75*), PRI (0.73*), WMI (0.56*) and PSI (0.84**) (see Table 8). Surprisingly, FASc children showed IGF-II negative correlations for all neuropsychological scores evaluated. This result indicates that the most affected children (FASc) have serious alterations in IGF-II metabolism and/or signalling pathways, suggesting that alternative pathways have to be activated in order to mitigate the neurological damage promoted by heavy alcohol exposure.

Moreover, IGF-I values showed a positive correlation with the anthropometric measurements height, weight and head circumference percentiles, especially for girls diagnosed with FASc or FASD.

Globally, correlations showed a significant effect of IGF-I over anthropometric measurements, in contrast to IGF-II which affected the neuropsychological variables.

## Discussion

In recent years, a large number of works have focused on finding biomarkers in order to improve the diagnosis of complex neurological diseases such as Alzheimer, Parkinson and various paediatric syndromes^22,23^. FASD represents one of the most complex diagnoses, based on physical and neurocognitive criteria^24^.

Growth retardation is a key element in FAS diagnosis and the analysis of IGF metabolism may be useful due to its role in pre- and postnatal growth^25,26^. Both IGF-I and IGF-II are detected in foetal circulation during early gestation, but their specific actions differ depending on the foetal tissue and gestational age. Altered prenatal expression of these growth factors and/or their receptors influences foetal growth^27–29^. However, although IGF-I and IGF-II exhibit a relevant function in pre- and postnatal growth mechanisms, their specific role in growth retardation in FASD children remains unclear. Furthermore, very limited data is available regarding the serum concentrations of these hormones among children and adolescents, especially for IGF-II.

The present study investigates the effects of PEE on distinct anthropometric and neuropsychological measurements and serum IGF-I/IGF-II concentrations. Child participants included: control (non-exposed to ethanol), PEE (no FASD diagnosis but meconium FAEE > 2 nmol/g) and FASD children adopted from EEC. The FASD group showed anthropometric variables such height, weight and BMI with values below the 10^th^ percentile. Despite of the relatively small sample size, IGF-II values in the 5^th^ percentile were significantly present in complete and partial FAS children and FASD IGF-I values were significantly lower than in the control group for both gender and the majority of age-range analysed. This decrease of postnatal serum IGF-I and IGF-II concentrations in FASD children adopted from Eastern Europe countries could be a biomarker of growth retardation deficiencies and could be considered as a biomarker of damage due to PEE.

These results are in line with previous reports^27,29,30^. Hellström *et al*. found that the median IGF-I and IGFBP-3 levels were in the low normal range in a small group of short children diagnosed with FAS^29^. Similarly, it has been suggested that infants of ethanol-abusing mothers may have low serum IGF-I levels^27^. Moreover, some authors have postulated that *IGF-1* gene polymorphisms are associated with reduced birth size^31^, and *IGF-1* receptor mutations have also been found in patients with intrauterine growth restriction^32^. IGF-II may influence placental function^28^ and a reduced placental expression of IGF-II is associated with intrauterine growth restriction^33^. In addition, Tunc-Ozcan *et al*. demonstrates that *IGF-2* expression is downregulated in the hippocampus after ethanol exposure during development^34^. Carter *et al*. have recently concluded that cognitive deficits generated by heavy PEE during foetal development correlate with growth restriction managed by IGF-I/IGF-II metabolism, suggesting that both could be used as FASD biomarkers^15^. Results from the current study are in contrast to Aros *et al*. who found a significant increase in IGF-I and IGF-II concentrations in a cohort of children exposed to ethanol^16^. However, this study showed a crucial limitation: they used a questionnaire instead of an objective biomarker to identify PEE children (FAEEs)^35,36^.

The present study also concludes a specific positive correlation between IGF-I values and anthropometric measures in girls diagnosed with FASD. These findings are concordant with previous studies^37^ which indicated greater sensitivity to brain damage in women exposed to alcohol and are consistent with the differences in IGF-I metabolic and signalling pathways depending on gender^38^.

Furthermore, IGF-II values correlate positively with neuropsychological scores for PEE and FASp children. However, the children most affected by alcohol exposure (FASc) show a clear negative correlation with neurological scores. These findings may indicate that an alternative neurological pathway could be activated to balance harmful effects of low IGF-II levels promoted by heavy alcohol exposure. Nevertheless, further studies have to be performed to analyse the molecular mechanism involved in the neurological recovery activated by low IGF-II values.

Alcohol alters the IGF metabolism in a tissue-specific manner which may contribute to different metabolic dysfunctions following prenatal alcohol exposure. This current study concludes that low IGF-I/IGF-II values are useful as FASD biomarkers due to the fact that low IGF-I values are related to morphological alterations and low IGF-II values correlate to neuropsychological dysfunctions in FASD children.

## Conclusion

In conclusion, IGF-I and IGF-II could be validated for FASD diagnosis as surrogate biomarkers of damage by prenatal alcohol exposure (related to growth retardation). To date, this is the first report which compared IGF levels on a paediatric population of FASD with children objectively non-exposed to ethanol with meconium FAEE < 2nmol/g, which is a relevant strength of the study. Future studies should evaluate the role of IGF-I and IGF-II as a potential biomarker of damage using bigger cohorts of patients objectively assessed for PEE.

## Patients and Methods

### Study participants and recruitment

Fifty-five children of the Meconium Project (28 boys and 27 girls) from 8 to 12 years old were prospectively recruited^36,39–43^. A prenatal assessment for drugs abuse and ethanol exposure was performed using FAEEs analysis in meconium, concluding that 44% of children were prenatally exposed to ethanol (meconium FAEE >2 nmol/g)^39^. Some of these children accepted to participate in the second part of the study 10 years later^12,44^. As previously reported^13^, the control group was established for FAEE concentration <2 nmol/g in meconium. Thirty-one children were considered negative for drugs of abuse and prenatal ethanol exposure (PEE) and 24 were positive to PEE (range of FAEE: from 2 to 248 nmol/g) but negative to drugs abuse.

Moreover, 98 children adopted from EEC (59 boys and 39 girls) from 3 to 18 years old, were recruited due to suspicion of FASD diagnosis (without any genetic condition). PEE was confirmed for all children adopted from EEC by adoption medical reports or adoption court rulings.

All methods were performed in accordance with the relevant guidelines and regulations. Informed consent from a parent and/or legal guardian for study participation was obtained. All protocols performed in this study were approved by the local ethical committee (CEIC-PSMAR: Comitè Ètic d’Investigació Clínica- Parc de Salut Mar) (2013/5272/I).

### Clinical evaluation

All children recruited in this study, including EEC adopted, PEE and non-exposed children (from the Meconium project) were independently evaluated by FAS dysmorphologists (O. García-Algar, MD and A. Bastons-Compta, MD) following the standardized dysmorphology exams for FASD diagnosis^45^. There was substantial agreement between the examiners for all dysmorphic features analysed. Paediatric examination included somatometric (height, weight and head circumference) and physical exams in order to evaluate minor abnormalities included in the characteristic FAS pattern such as palpebral fissure length, upper lip, philtrum, railway ears, midface hypoplasia, and stick hockey hands. Height, weight, head circumference and palpebral fissure length were converted to standard deviation (SD) scores. Underweight, short stature, microcephaly and short palpebral fissure were defined as values ≤2 SD below the reference scales or ≤10^th^ percentile. Upper lip and philtrum were scored by using a ruler and the “lip-philtrum guide” (thin upper lip and smooth philtrum were defined as a score of 4 or 5)^24^.

### Neurocognitive assessment

The Wechsler Intelligence Scale for Children, Fourth Edition (WISC-IV)^46^ was used to obtain a full-scale IQ (FSIQ) score as well as composite index scores for the following individual cognitive domains: Verbal Comprehension Index (VCI), which measures verbal concept formation, verbal reasoning, and knowledge acquired from one’s environment; Perceptual Reasoning Index (PRI), which measures perceptual and fluid reasoning, spatial processing and visual-motor integration; Working Memory Index (WMI), which requires working memory processes to manipulate orally presented verbal sequences; and Processing Speed Index (PSI), which requires visual perception and organization, visual scanning and the ability to to coordinate the use of hands and eyes effeciently. WISC-IV index standard scores have a mean of 100 and a standard derivation of 15. For the study, neurodevelopment test was considered delayed if the composite domain scores were <70, for three or more domains. Tests were administered in the language of instruction used in the child’s classroom.

### FASD diagnosis

Each child was assigned to a FASD diagnostic category according to the clarification of the 1996 IOM criteria (reviewed on 2005^24^). Data was gathered for 5 diagnostic characteristics: [1] confirmed prenatal alcohol exposure; [2] evidence of a characteristic minor facial abnormalities pattern, typified by having a thin upper lip, smooth philtrum and short palpebral fissures (two of them at least); [3] growth retardation, defined as prenatal and/or postnatal height or weight ≤10th percentile; [4] evidence of deficient brain growth; and [5] behavioural or cognitive abnormalities related to prenatal alcohol exposure. For a diagnosis of complete FAS, at least criterion 1, 2, 3, 4 (confirmed prenatal alcohol exposure) or 2, 3, 4, 5 (no confirmed prenatal alcohol exposure) were required; for partial FAS, criterion 1, 2, and at least one of criteria 3, 4, 5 (confirmed prenatal alcohol exposure) or 2 and at least one of criteria 3, 4, or 5 (no confirmed prenatal alcohol exposure) were required. The diagnosis of alcohol-related birth defects (ARBD) required the detection of criteria 1 and 2, plus a minimum of one structural defect involving heart, skeleton, kidney, eye, ear or minor abnormalities like railway ears, midface hypoplasia or stick hockey hands. The diagnosis of alcohol-related neurodevelopmental disorders (ARND) required the detection of criteria 1, 4, and/or 5.

### Serum IGF-I and IGF-II determination

Serum concentrations of IGF-I and IGF-II were determined in all children. Serum IGF-I levels were analysed by immunoassay (REF: DG100; R&D Systems, Minneapolis, MN). The sensitivity of this assay was 0.026 ng/ml, intra-assay CV was 4.03%, and the inter-assay CV was 7.97%. IGF-II serum concentrations were determined using a commercial immunoassay (REF: RMEE30; Biovendor, Düsseldorf, Germany) validated for *in vitro* diagnostic use in the European Union. The sensitivity of the assay was 0.02 ng/ml, intra-assay CV was 4.84% and the inter-assay CV was 7.11%. The reference values of the IGF-I hormone for children (distributed for age and gender) come from the Nichols Institute Diagnostica^20^. IGF-II reference values were supplied by the immunoassay kit provider.

### Statistical analysis

Database management and statistical analysis were performed using SPSS v.22 (IBM, Armonk, NY) and GraphPad software 6.0. MedCalc 16 and GraphPad software 6.0 was used for Spearman (r) correlation analysis. Descriptive statistics were performed using mean, Standard Deviation (SD) and error (Std. Error). Linear regression and percentiles distribution were obtained according to the reference values concluded in previous studies. *t-test* was used to compare the distribution of age and gender for the different groups, using Holm-Sidak correction. In addition, the outcomes of the anthropometric measurements and the neurocognitive assessments for FASD, PEE and non-exposed children were compared using *t-test* analysis as well. Non-parametric Dunn’s multiple comparison tests were used to study the differences among control, PEE and FASD (FASc, FASp, ARNB, ARBD) groups for IGF-I and IGF-II values. Linear regression was developed to analyse the relevance of age and gender in IGF-I and IGF-II serum concentrations. Levels of IGF-I between reference values and FASD, PEE or non-exposed children were evaluated by *t-tests*. Reference values were used to compare the <5^th^ and <50^th^ percentiles referred to IGF-II values among FASD, PEE and non-exposed categories. Statistical significance was set at *p-*value <0.05 for all analyses performed.

### What’s known on This Subject?

Alcohol is the main human teratogen and its consumption during pregnancy can cause a spectrum of malformations and cognitive impairment called Foetal Alcohol Spectrum Disorders.

### What This Study Adds

IGF-I and IGF-II can be considered biomarkers of prenatal exposure to ethanol and diagnostic markers of Foetal Alcohol Syndrome.
